# Rapid cardiovascular aging following allogeneic hematopoietic cell transplantation for hematological malignancy

**DOI:** 10.3389/fcvm.2022.926064

**Published:** 2022-12-15

**Authors:** Hayley T. Dillon, Stephen Foulkes, Yuki A. Horne-Okano, David Kliman, David W. Dunstan, Robin M. Daly, Steve F. Fraser, Sharon Avery, Bronwyn A. Kingwell, Andre La Gerche, Erin J. Howden

**Affiliations:** ^1^Clinical Research Domain, Baker Heart and Diabetes Institute, Melbourne, VIC, Australia; ^2^Institute of Physical Activity and Nutrition, School of Exercise and Nutrition Sciences, Deakin University, Geelong, VIC, Australia; ^3^Malignant Haematology and Stem Cell Transplantation Service, Alfred Hospital, Melbourne, VIC, Australia; ^4^CSL Ltd, Melbourne, VIC, Australia

**Keywords:** cardiac function, cardiopulmonary fitness, hematological cancer, exercise testing, cardiotoxicity, cardiovascular disease

## Abstract

**Introduction:**

Allogeneic hematopoietic cell transplantation (allo-HCT) offers a potential cure for high-risk hematological malignancy; however, long-term survivors experience increased cardiovascular morbidity and mortality. It is unclear how allo-HCT impacts cardiovascular function in the short-term. Thus, this 3-month prospective study sought to evaluate the short-term cardiovascular impact of allo-HCT in hematological cancer patients, compared to an age-matched non-cancer control group.

**Methods:**

Before and ~3-months following allo-HCT, 17 hematological cancer patients (45 ± 18 years) underwent cardiopulmonary exercise testing to quantify peak oxygen uptake (VO_2_peak)—a measure of integrative cardiovascular function. Then, to determine the degree to which changes in VO_2_peak are mediated by cardiac vs. non-cardiac factors, participants underwent exercise cardiac MRI (cardiac reserve), resting echocardiography (left-ventricular ejection fraction [LVEF], global longitudinal strain [GLS]), dual-energy x-ray absorptiometry (lean [LM] and fat mass [FM]), blood pressure (BP) assessment, hemoglobin sampling, and arteriovenous oxygen difference (a-vO_2_diff) estimation *via* the Fick equation. Twelve controls (43 ± 13 years) underwent identical testing at equivalent baseline and 3-month time intervals.

**Results:**

*S*ignificant group-by-time interactions were observed for absolute VO_2_peak (*p* = 0.006), bodyweight-indexed VO_2_peak (*p* = 0.015), LM (*p* = 0.001) and cardiac reserve (*p* = 0.019), which were driven by 26, 24, 6, and 26% reductions in the allo-HCT group (all *p* ≤ 0.001), respectively, as no significant changes were observed in the age-matched control group. No significant group-by-time interactions were observed for LVEF, GLS, FM, hemoglobin, BP or a-vO_2_diff, though a-vO_2_diff declined 12% in allo-HCT (*p* = 0.028).

**Conclusion:**

In summary, allo-HCT severely impairs VO_2_peak, reflecting central and peripheral dysfunction. These results indicate allo-HCT rapidly accelerates cardiovascular aging and reinforces the need for early preventive cardiovascular intervention in this high-risk group.

## Introduction

Hematological malignancies accounted for 1.28 million (6.6%) and 711, 840 (7.1%) cancer diagnoses and deaths globally in 2020 (1). Accordingly, allogeneic hematopoietic cell transplantation (allo-HCT) rates to manage these malignancies have more than doubled between 2006–2016 (2). This increase in allo-HCT, combined with advances in human leukocyte antigen-matched donor selection, graft-vs.-host disease (GvHD) prevention and management, and supportive care have contributed to a progressive growth in long-term cancer survivors (3, 4). However, the curative potential of allo-HCT continues to be offset by significant cardiovascular morbidity and mortality. Indeed, compared to age-matched non-cancer controls, long-term allo-HCT survivors (≥2 y) experience elevated rates of cardiovascular disease (CVD) (5–9) and serious cardiovascular events (9–11), culminating in a 2-to-4-fold increased risk of premature cardiovascular mortality (11, 12). These data provide compelling evidence of an accelerated cardiovascular aging phenotype among allo-HCT survivors and have sparked a call for studies aimed at understanding allo-HCT-induced CVD in order to inform efficacious preventive intervention (13).

The paradigm explaining the deterioration in cardiovascular health among allo-HCT survivors suggests there are multiple contributing factors including: the cancer itself (14); anti-cancer therapies (15); prolonged bedrest resulting in muscle loss and physical deconditioning (16); and the inflammatory perturbations of allografting which is exacerbated by GvHD and its prophylaxis/treatment (17, 18). Importantly, evidence extrapolated from studies with overlapping exposures suggests these insults are particularly deleterious to the heart (15–17), but also impact the entire cardiovascular-hematological-skeletal muscle axis (15–21). However, despite evidence of accelerated cardiovascular aging in long-term allo-HCT survivors (e.g., premature onset of overt CVD and related mortality), few studies have prospectively characterized the short-term cardiovascular impact of allo-HCT. Further, the cardiovascular impact of allo-HCT has been minimally characterized using sensitive biomarkers or state-of-the-art, high-resolution physiological testing at any point of the allo-HCT survivorship continuum. Therefore, the clinical trajectory and pathogenesis of allo-HCT related CVD remains unclear—two factors integral for informing the design (e.g., type and timing) of efficacious cardiovascular intervention.

Early detection of cancer treatment-related cardiac dysfunction is critical to facilitate prompt intervention and more effectively prevent irreversible damage and long-term morbidity. Accordingly, the application of exercise stress for the quantification of cardiovascular reserve (defined as the increase in cardiovascular function from rest to peak exercise) has emerged as an efficacious approach in unmasking subclinical cardiovascular pathology (22–24), and predicting all-cause, cardiovascular, and cancer-specific mortality (25–27). Cardiovascular reserve can be evaluated *via* a specific approach using exercise cardiac magnetic resonance imaging (exercise CMR) to directly quantify cardiac reserve (ability to augment cardiac output during exercise), or an integrative approach using cardiopulmonary exercise testing (CPET) for the assessment of peak oxygen uptake (VO_2_peak). Importantly, beyond capturing cardiac reserve, VO_2_peak also encapsulates the integrative function of non-cardiac, “peripheral” organ systems (hematological, vascular, skeletal muscle), which play an important role in the pathogenesis and pathophysiology of CVD (28–31), and are postulated to be impaired by allo-HCT. Hence, early cardiovascular follow-up with exercise-based measures that can provide a more accurate and comprehensive characterization of central and peripheral organ functioning may aid in guiding improved diagnostic and therapeutic approaches necessary to prevent long-term cardiovascular morbidity in this high-risk patient group.

Therefore, this 3-month prospective study sought to evaluate the short-term cardiovascular impact of allo-HCT, assessed primarily as VO_2_peak and cardiac reserve, with direct comparison to an untreated age-matched non-cancer control group.

## Methods

### Study population and design

We performed a prospective cohort study comparing adults with hematological cancer scheduled for allo-HCT and age-matched non-cancer controls. Allo-HCT patients were recruited *via* direct referral from the Alfred Health HCT coordinators in Melbourne, Australia. Controls were recruited from the community who responded to advertisements seeking ostensibly healthy adults. Exclusion criteria for both groups included: (1) age <18 years, (2) inability to speak/understand English, and (3) known contraindications to CPET or CMR (i.e., injury, pacemaker, implanted metallic foreign body or device). Additional exclusion criteria for controls included: (1) BMI ≥35 kg.m^−2^, (2) presence of a significant underlying medical condition(s), and (3) participation in ≥150-min of moderate intensity or ≥75-min of vigorous intensity aerobic physical activity per week.

### Study protocol and experimental measurements

Participants underwent a comprehensive battery of physiological testing on two occasions. The allo-HCT group underwent testing prior to [median [IQR], 16 (11–27) days], and ~3-months following allo-HCT, while controls underwent identical testing, on two time-points, ~3-months apart. Participants were asked to refrain from moderate to vigorous intensity physical activity in the 24-h preceding testing and abstain from alcohol and caffeine on the day of testing.

#### Participant medical history

A complete medical history of the allo-HCT patients was obtained from the Alfred Health Clinical Database and information relating to diagnosis, cardiovascular risk profile, prior treatment history, allo-HCT (donor, graft source, conditioning intensity, GvHD prophylaxis, GvHD status) and current medication use were recorded. A general lifestyle questionnaire was administered to controls to obtain information relating to current health status and relevant medical history including use of any medications.

#### Cardiopulmonary fitness

An incremental ramp protocol CPET was conducted on an electronically braked cycle ergometer (Lode Excalibur Sport, Groningen, the Netherlands) for the measurement of VO_2_peak. Briefly, participants cycled at 10–25 Watts for 1-min, after which, the workload increased at a progressive rate of 10–30 Watts.min^−1^ until volitional fatigue. The ramp protocol was individualized according to participant age, weight, self-reported exercise capacity, and physical activity history, with the intention of achieving volitional fatigue within 8-to-12-min. Breath-by-breath expired air gas analysis was performed continuously throughout testing using a calibrated metabolic cart (Vyntus CPX, Carefusion, San Diego, USA). Blood pressure (BP) was measured at 2-min intervals using an ECG-gated electrosphygmomanometer BP cuff (Tango M2 Stress Test Monitor and Orbit-K Blood Pressure Cuff, SunTech Medical Inc. Morrisville, USA). Heart rate (HR) and electrical activity were monitored continuously with a 12-lead electrocardiogram (Vyntus^TM^ ECG 12-lead PC-ECG, Vyaire Medical, Mettawa, USA). VO_2_peak was defined as the average of the six consecutive highest 5-sec VO_2_ values, and percent of age-, height-, weight and sex-predicted VO_2_peak was calculated according to the FRIEND reference equation (32). V_E_/VCO_2_ was assessed from linear regression of V_E_ and VCO_2_ values as it has been validated as an important prognostic marker in patients with heart failure, independent of VO_2_peak (33). Contraindications to CPET adhered to the American Thoracic Society recommendations (34). In addition, a lower limit of 80 g.L^−1^ of hemoglobin was employed in line with clinical hemoglobin transfusion thresholds.

#### Resting cardiac function

Resting cardiac function was evaluated *via* echocardiogram (Vivid E95, General Electric Medical Systems, Milwaukee, Wisconsin). Images were collected, saved in a digital format, and analyzed offline (Echopac v13.0.00, GE, Norway) by a trained sonographer. A three-dimensional full-volume dataset was acquired to measure left-ventricular ejection fraction (LVEF). Two-dimensional speckle tracking echocardiography-derived global longitudinal strain (GLS) was quantified from three apical views at a temporal resolution of 60–90 frames.sec^−1^ with GLS defined as the average negative value of the strain rate curves.

#### Peak cardiac function and cardiac reserve

The biventricular response to exercise was evaluated using a validated real-time CMR method (35). Exercise was performed within the CMR bore using an electronically braked supine cycle ergometer (MR Ergometer Pedal, Lode, Groningen, the Netherlands). Cardiac images were acquired using a Siemens MAGNETOM Prisma 3.0T CMR with a five-element phased array coil at rest and during exercise at 60% of the maximal power output achieved during CPET as this approximates maximal exercise capacity in supine (35). Real-time steady-state free-precession cine MR imaging was performed without cardiac or respiratory gating at a temporal resolution of 36–38 ms and a three-dimensional stack of 10–18 adjoining 8-mm image slices, encapsulating both ventricle and atria, were acquired in the short axis (SAX) and horizontal long-axis (HLA) planes.

Real-time cine images were analyzed offline in RightVol (KUL, Leuven, Belgium). End-diastole and end-systole were retrospectively marked at end-expiration, and the left- and right-ventricular endocardia (papillary muscles and trabeculations included in the blood pool) were manually contoured on the SAX images, with reference to the atrioventricular valve plane in the HLA. Ventricular volumes were quantified at rest and peak exercise *via* the summation of disc method. Stroke volume index (SVI) was calculated as the difference between end-diastolic volume and end-systolic volume, indexed to body surface area (BSA), while cardiac index (CI) was calculated as stroke volume multiplied by HR, indexed to BSA. Left- and right-ventricular ejection fractions (LVEF, RVEF) were calculated as SV/end-diastolic volume, multiplied by 100. Cardiac (CI, SVI, HR) and contractile (LVEF, RVEF) reserve were defined as the ability to augment cardiac function from rest to peak exercise (peak values—rest values). Arteriovenous oxygen difference (a-vO_2_diff) was estimated *via* the Fick equation using CPET-derived VO_2_peak and exercise CMR-derived peak cardiac output.

#### Biochemistry

Blood samples were collected in the morning after an overnight fast to measure hemoglobin concentration, cardiac Troponin-I (cTn-I) and B-natriuretic peptide (BNP).

#### Anthropometry and body composition

Height (m) and body mass (kg) were assessed and used to calculate BMI and BSA. Total lean mass (LM, kg), fat mass (FM, kg) and percentage body fat (%BF) were quantified using dual-energy X-ray absorptiometry (GE Lunar iDXA, GE Healthcare, Little Chalfont, UK), with scans manually analyzed using enCore software (version 14.10.022).

#### Blood pressure

After resting in the supine position for 10-min in a quiet room, resting BP and HR were measured in triplicate at the brachial artery using an automated oscillometric BP monitor (OMRON HEM-907, OMRON Corporation, Tokyo, Japan). The average of three measurements was used for analysis.

#### Definitions of cardiotoxicity and functional disability

Cardiotoxicity was defined using standard echocardiography criteria (15): (1) an absolute reduction in LVEF of >15%, to a value >50%, (2) an absolute reduction in LVEF of >10%, to a value <50% and (3) a >12% relative reduction in GLS. Functional disability was defined as VO_2_peak <18 ml.kg^−1^.min^−1^ as per the American Heart Association Scientific Statement (36).

### Sample size

The sample size calculation for the allo-HCT group was based on the reported reduction in VO_2peak_ following 4-weeks of bedrest in healthy individuals (37). Indeed, the pooling of 19 bedrest investigations suggests %Δ VO_2_peak can be explained by the linear regression = 1.4–0.85 (days), r = −0.73 (37). Considering the average hospital stay for allo-HCT patient is 28 days, a ~22.4% reduction in VO_2_peak was expected. To account for normal variation in test-retest reproducibility (4.4%), the study was powered to detect an 18% difference between allo-HCT and non-cancer control groups. With estimated standard deviation of 12% (obtained from our study in women treated for breast cancer), 15 allo-HCT and 8 non-cancer control completions were deemed necessary (SD = 12%; 90% power; alpha = 0.05). Sample size was increased ~20% to account for possible drop-out.

### Statistical analysis

Analysis was performed using SPSS software (version 24.0, Statistical Package for the Social Sciences, IBM, Chicago, USA). Continuous data were inspected for normality, linearity and homoscedasticity and presented as mean ± SD or mean (95% CI). Categorical data are presented as *n* (%). Independent *t*-tests or Fishers exact tests were performed to assess baseline group differences for continuous and categorical variables, respectively. Treatment effects were assessed *via* generalized linear mixed modeling with covariance structure informed by the Akaike information criteria. The model included time as the repeated measure, group and group-by-time as fixed effects, and participants as random effects. Findings remained unchanged when adjusting for sex, thus, unadjusted results are presented. Within-group changes after 3-months are expressed as mean (95% CI) change from baseline and between-group differences for the mean changes after 3-months [net difference (95% CI)] were calculated by subtracting the within-group changes from baseline for controls from the within-group changes for allo-HCT. CTn-I was transformed to yield a normal distribution before analysis. Two-sided *p* < 0.05 indicated significance.

## Results

### Participant characteristics and transplant-related information

Twenty-six individuals scheduled for allo-HCT (17 men, 9 women) and 12 age-matched non-cancer controls (5 men, 7 women) were recruited and completed all baseline assessments. After 3-months, all controls (100%) and 17 (65%; 12 men, 5 women) allo-HCT participants completed follow up (*n* = 7 deceased, *n* = 1 declined due to perceived incapacity, *n* = 1 lost-to-follow-up) and were included in analyses. There were no significant differences in baseline participant characteristics or transplant-related factors between allo-HCT recipients who did and did not complete follow-up (see Supplementary Table S1).

Characteristics of the allo-HCT and control participants who completed follow-up are summarized in Tables 1, 2. There were no significant differences in demographic, anthropometric or traditional cardiovascular risk factors between allo-HCT recipients and controls (Table 1). However, allo-HCT recipients had a significantly lower VO_2_peak (*p* < 0.001), cardiac reserve (*p* = 0.004), LVEF (*p* = 0.043), and GLS (*p* = 0.018), relative to controls (Table 1). Acute myeloid leukemia was the most common transplant indication (65%) among allo-HCT recipients, and treatment history was diverse (A more detailed summary of prior treatments is provided in Supplementary Table S1). Sixteen participants (94%) had previous chemotherapy exposure, with anthracycline and anti-metabolite agents most frequently administered (both 82%). With regard to transplant related factors, the most common donor type, graft source, conditioning intensity and GvHD prophylaxis were unrelated, peripheral blood, reduced intensity, and methotrexate/ciclosporine, respectively (Table 2).

**Table 1:** Baseline characteristics for Allo-HCT and Control participants who completed baseline and follow-up assessments.

	**Allo-HCT**	**Control**
	**(*n* = 17)**	**(*n* = 12)**
**Sex, % male**	70%	42%
**Age, years**	45 ± 18	43 ± 13
**Weight, kg**	80.7 ± 18.0	74.3 ± 14.4
**Body mass index, kg.m** ^**−2**^	27.4 ± 6.2	24.6 ± 3.7
**Cardiovascular function**		
LVEF, %	54.7 ± 5.5*	59.5 ± 5.7*
GLS, %	−17.8 ± 2.0*	−20.0 ± 2.4
CI Reserve, L.min^−1^.m^−2^	3.8 ± 1.4**	5.9 ± 1.7
VO_2_peak, ml.kg^−1^.min^−1^	22.9 ± 8.0***	34.8 ± 8.1
VO_2_peak, % predicted	67 ± 12***	104 ± 16
Functional disability, *n* (%)	5 (29)	0 (0)
**Cardiovascular risk factors**, ***n (%)***		
Hypertension	4 (24)	0 (0)
Hyperlipidaemia	2 (12)	0 (0)
Diabetes	1 (6)	0 (0)
Body mass index ≥25 kg.m^−2^	9 (53)	5 (42)
Previous cardiovascular event	2 (12)	0 (0)
≥1 cardiovascular risk factor	10 (59)	5 (42)
**Cardiovascular medications**, ***n (%)***		
Statin/Cholesterol absorption inhibitor	1 (6)	0 (0)
Antihypertensives	2 (12)	0 (0)
Beta–blocker	1 (6)	0 (0)
Antidiabetic	1 (6)	0 (0)
Non–steroidal anti–inflammatory	3 (18)	1 (8)
**Diagnosis**, ***n*** **(%)**		
Acute myeloid leukemia	11 (65)	n/a
Non–Hodgkin lymphoma	3 (18)	n/a
Acute lymphoblastic leukemia	2 (12)	n/a
Myelodysplasia	1 (6)	n/a
**Prior cancer treatment**, ***n (%)***		
No prior treatment	1 (6)	n/a
Chemotherapy	16 (94)	n/a
Cumulative anthracycline dose, mg.m^−2^	180 (100−270)	n/a
Targeted Therapy	5 (29)	n/a
Immunotherapy	3 (18)	n/a
Radiation	2 (12)	n/a
Autologous stem cell transplant	1 (6)	n/a

Data are mean ± SD, median (IQR) or n (%). CI, cardiac index; GLS, global longitudinal strain; LVEF, left-ventricular ejection fraction; VO_2_peak, peak oxygen uptake. *p < 0.05, **p < 0.01, ***p < 0.001 vs. control.

**Table 2:** Transplant related information for Allo-HCT participants that completed baseline and follow–up assessments.

	***N* (%)**
**Donor type**	
Related	8 (47)
Unrelated	9 (53)
**Graft source**	
Bone marrow	3 (18)
Peripheral blood stem cell	14 (82)
**Conditioning intensity**	
Myeloablative	7 (41)
Reduced Intensity	10 (59)
**Conditioning regimen**	
Ciclosporin/TBI	6 (35)
Flu/Mel	6 (35)
Flu/Mel/Campath	3 (18)
ETP/TBI	1 (6)
LACE	1 (6)
**GvHD prophylaxis**	
MTX/Ciclosporin ± ATG	10 (59)
PTCy/Ciclosporin	4 (24)
Ciclosporin	2 (12)
TAC	1 (6)
**Acute GvHD grade**	
No GvHD	10 (59)
Grade I	6 (35)
Grade II	1 (6)
Hospital length of stay, days	31 (21–39)

Data are n (%) or median (range). ATG, ATGAM thymoglobulin; ETP, Etoposide; Flu, Fludarabine; GvHD, Graft–vs.–host disease; LACE, Lomustine, Cytarabine, Cyclophosphamide, Etoposide; Mel, Melphalan; MTX, Methotrexate; PTCy, Post–transplant Cyclophosphamide; TAC, Tacrolimus; TBI, Total body irradiation.

### Exercise capacity

**Figure 1:**
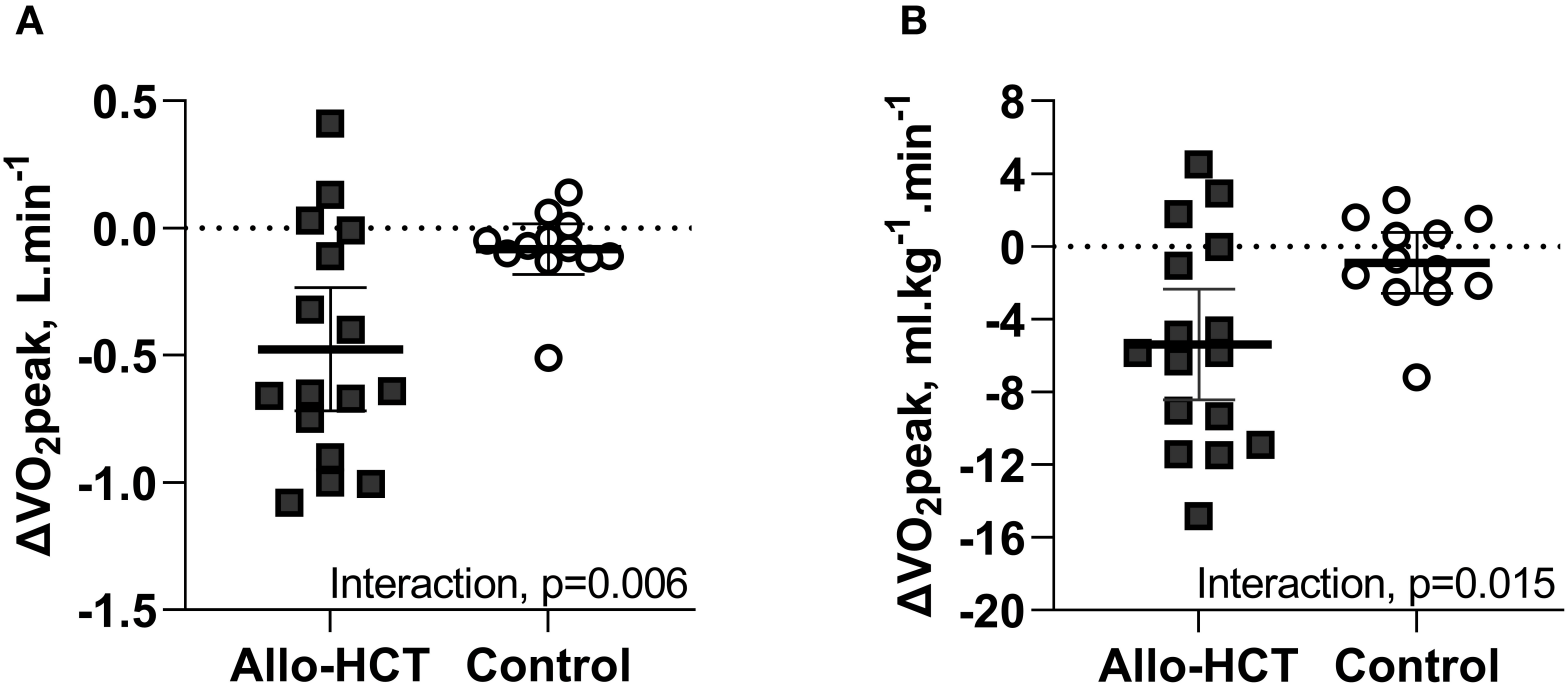
**(A,B)** Mean (95% CI) 3-month change from baseline in absolute and bodyweight-indexed VO_2_peak in Allo-HCT and Control assessed by cardiopulmonary exercise testing. There was a significant between-group difference for the net change from baseline for absolute and bodyweight-indexed VO_2_peak (*p* = 0.006 and *p* = 0.015, respectively), which was due to a significant decrease in allo-HCT and no change in controls.

As shown in Figures 1A,B, a significant between-group difference existed for the net change over 3-months for absolute (−0.4 L.min^−1^ [95% CI −0.7, −0.1]; group-by-time-interaction, *p* = 0.006) and bodyweight-indexed VO_2_peak (−4.5 ml.kg^−1^.min^−1^ [95% CI −8.1, −0.9]; group-by-time interaction, *p* = 0.015), due to a 26% (−0.5 L.min^−1^ [95% CI −0.7, −0.3]; *p* < 0.001) and 24% (−5.4 ml.kg^−1^.min^−1^ [95% CI −7.7, −3.1]; *p* < 0.001) decline in allo-HCT recipients, respectively, and no significant change in controls. Consequently, allo-HCT recipients achieved a follow-up VO_2_peak that was, on average, 49% below predicted (-16% from baseline; *p* < 0.001), with 53% considered functionally disabled. As shown in Table 3, significant between-group differences (group-by-time interactions) were also observed for peak power output (*p* < 0.001) and percentage of age-predicted HR_peak_ (*p* = 0.005), which was driven by a 30% and 11% reduction from baseline in the allo-HCT recipients (both *p* < 0.001) as no significant change was observed in controls. A similar significant group-by-time interaction was noted for V_E_/VCO_2_ slope (*p* = 0.008), which was due to a 17% increase in allo-HCT recipients (*p* < 0.001) as there was no change in controls. Peak a-vO_2_diff declined 12% in allo-HCT recipients (*p* = 0.028), but a significant between-group difference for the net change from baseline was not observed (interaction, *p* = 0.23), due to a slight, non-significant downward shift in controls.

**Table 3:** Mean baseline values, within-group changes after 3–months and the net between-group differences for the change for peak CPET parameters in Allo-HCT and Control groups.

**Measure**	**Allo-HCT**	**Control**	**Δ net difference**	**Group**,	**Time**,	**Group x Time**,
			**(HCT vs. Con)**	**p**	**p**	**p**
**VO**_**2**_ **peak, L.min** *^**−**^^1^*
Baseline	1.8 ± 0.6	2.6 ± 0.7				
3–months	1.3 ± 0.4	2.5 ± 0.7				
Δ	−0.5 (−0.7, −0.3)***	−0.1 (−0.2, 0.1)	−0.4 (−0.7, −0.1)	<0.001	<0.001	0.006
**VO**_**2**_ **peak, ml.kg** ^**−1**^ **.min** ^**−**^ *^1^*
Baseline	22.9 ± 8.0	34.8 ± 8.1				
3–months	17.5 ± 5.7	33.9 ± 8.0				
Δ	−5.4 (−7.7, −3.1)***	−0.9 (−3.6, 1.8)	−4.5 (−8.1, −0.9)	<0.001	0.001	0.015
**VO**_**2**_ **peak, % predicted**
Baseline	67 ± 12	104 ± 16				
3–months	51 ± 13	101 ± 17				
Δ	−16 (−23, −10)***	−3 (−11, 4)	−13 (−23, −3)	<0.001	<0.001	0.012
**Peak Power Output, Watts**
Baseline	154 ± 62	254 ± 68				
3–months	108 ± 44	258 ± 69				
Δ	−46 (−59, −32)***	4 (−12, 19)	−50 (−70, −29)	<0.001	<0.001	<0.001
**HR**_**peak**_ **, % predicted**
Baseline	96 ± 8	102 ± 4				
3–months	85 ± 11	101 ± 4				
Δ	−11 (−15, −6)***	−1 (−6, 5)	−10 (−17, −3)	<0.001	0.002	0.005
**Peak RER**
Baseline	1.38 ± 0.09	1.33 ± 0.15				
3–months	1.31 ± 0.14	1.33 ± 0.07				
Δ	−0.07 (−0.13, −0.01)*	0.00 (−0.07, 0.07)	−0.07 (−0.16, 0.02)	0.79	0.13	0.15
**V**_**E**_**/VCO**_**2**_ **slope**
Baseline	27.8 ± 3.4	26.8 ± 2.9				
3–months	32.5 ± 5.5	26.5 ± 4.0				
Δ	4.7 (2.3, 7.1)***	−0.3 (−3.0, 2.4)	5.0 (1.4, 8.6)	0.011	0.017	0.008
**Peak a–vO**_**2**_ **diff, %**
Baseline	12.0 ± 2.6	14.7 ± 1.7				
3–months	10.5 ± 2.7	14.4 ± 1.9				
Δ	−1.5 (−2.8, −0.2)*	−0.3 (−1.7, 1.0)	−1.1 (−3.0, 0.7)	<0.001	0.06	0.23

All baseline and 3–month values are unadjusted means ± SDs; all within-group changes are unadjusted mean (95% CI) and expressed as absolute change from baseline. Mean net differences were calculated by subtracting the within–group changes from baseline in the control group from the within–group change from baseline in the allo-HCT group after 3 months. *p < 0.05, ***p < 0.001 vs. baseline. VO_2_peak, peak oxygen uptake; HR_peak_, peak heart rate; RER, respiratory exchange ratio; peak a–vO_2_diff, peak arteriovenous oxygen difference.

### Resting cardiac function and cardiac biomarkers

Echocardiographic measures of resting cardiac function and cardiac biomarkers were unchanged in both groups at 3-months (Table 4). No participants developed overt CVD or met LVEF cardiotoxicity criteria, but one allo-HCT recipient commenced treatment for arrhythmia, and three allo-HCT recipients and two controls had clinically significant GLS declines. One allo-HCT recipient had a post-treatment troponin >15 ng.L^−1^ and two had a post-treatment BNP >100 ng.L^−1^, totalling a 35% incidence of subclinical cardiac pathology in allo-HCT and 17% in controls (between-group difference, *p* = 0.41).

**Table 4:** Mean baseline values, within-group changes after 3–months and the net between-group differences for the change for cardiac parameters in Allo-HCT and Control groups.

**Measure**	**Allo-HCT**	**Control**	**Δ net difference**	**Group**,	**Time**,	**Group x Time**,
			**(HCT vs. Con)**	**p**	**p**	**p**
Cardiac biomarkers	
**cTn–I, ng.L** ^**−1**^	
Baseline	4.6 ± 4.2	2.8 ± 1.2				
3–months	5.4 ± 5.7	2.9 ± 1.1				
Δ	0.8 (−1.8, 3.4)	0.1 (−2.8, 3.0)	0.71 (−3.2, 4.6)	0.14	0.63	0.72
**BNP, ng.L** ^**−**^ *^1^*	
Baseline	40.5 ± 32.0	33.2 ± 22.9				
3–months	54.3 ± 35.6	31.7 ± 28.5				
Δ	13.8 (−3.2, 30.8)	−1.6 (−21.2, 18.1)	15.4 (−10.6, 41.3)	0.22	0.34	0.24
Resting echocardiography		
**LVEF, %**		
Baseline	54.7 ± 5.5	59.5 ± 5.7				
3–months	56.1 ± 3.8	57.6 ± 4.4				
Δ	1.4 (−1.3, 4.1)	−1.8 (−4.9, 1.3)	3.2 (−0.9, 7.3)	0.065	0.84	0.12
**GLS, %**		
Baseline	−17.8 ± 2.0	−20.0 ± 2.4				
3–months	−17.3 ± 1.5	−19.4 ± 1.9				
Δ	0.5 (−0.6, 1.6)	0.6 (−0.7, 2.0)	−0.1 (−1.8, 1.6)	0.001	0.18	0.90
Resting CMR	
**HR, beats.min** ^**−1**^	
Baseline	81 ± 12	66 ± 9				
3–months	84 ± 6	63 ± 9				
Δ	3 (−5, 10)	−3 (−11, 5)	6 (−5, 16)	<0.001	0.98	0.30
**SVI, ml.m** ^**−2**^	
Baseline	50.0 ± 7.4	54.2 ± 9.2				
3–months	47.6 ± 9.0	53.7 ± 8.3				
Δ	−2.4 (−5.2, 0.3)	−0.5 (−3.4, 2.4)	−1.9 (−5.9, 2.1)	0.14	0.14	0.33
**CI, L.min** ^**−1**^ **.m** ^**−2**^	
Baseline	4.0 ± 0.8	3.6 ± 0.6				
3–months	4.0 ± 0.9	3.4 ± 0.5				
Δ	0.0 (−0.4, 0.4)	−0.2 (−0.6, 0.2)	0.2 (−0.4, 0.7)	0.032	0.42	0.58
**LVEF, %**	
Baseline	53.4 ± 5.5	56.5 ± 2.6				
3–months	53.2 ± 4.3	56.2 ± 3.2				
Δ	−0.2 (−3.2, 2.8)	−0.3 (−3.4, 2.8)	0.1 (−4.2, 4.4)	0.026	0.80	0.96
**RVEF, %**	
Baseline	55.5 ± 3.7	52.4 ± 4.7				
3–months	55.6 ± 2.9	52.8 ± 4.3				
Δ	0.1 (−1.9, 2.1)	0.5 (−1.6, 2.6)	−0.3 (−3.2, 2.5)	0.054	0.67	0.81
Exercise CMR	
**HR**_**peak**_ **, beats.min** ^**−1**^	
Baseline	136 ± 16	147 ± 13				
3–months	130 ± 16	146 ± 14				
Δ	−6 (−12, 1)	−2 (−8, 6)	−4 (−14, 5)	0.029	0.14	0.37
**SVI**_**peak**_ **, ml.m** ^**−2**^	
Baseline	57.9 ± 10.0	63.8 ± 11.6				
3–months	52.9 ± 11.0	63.2 ± 9.6				
Δ	−5.0 (−8.1, −1.9)**	−0.6 (−3.9, 2.7)	−4.4 (−8.9, 0.2)	0.063	0.017	0.058
**CI**_**peak**_ **, L.min** ^**−1**^ **.m** ^**−2**^	
Baseline	7.8 ± 1.5	9.4 ± 2.1				
3–months	6.8 ± 1.3	9.2 ± 1.7				
Δ	−1.0 (−1.5, −0.5)***	−0.2 (−0.8, 0.2)	−0.8 (−1.6, 0.0)	0.004	0.002	0.042
**LVEF**_**peak**_ **, %**	
Baseline	59.2 ± 4.3	64.1 ± 3.7				
3–months	57.3 ± 5.4	64.1 ± 3.6				
Δ	−1.9 (−3.6, −0.2)*	0.0 (−1.8, 1.8)	−1.9 (−4.4, 0.6)	0.001	0.13	0.14
**RVEF**_**peak**_ **, %**	
Baseline	62.4 ± 3.0	61.9 ± 5.5				
3–months	59.2 ± 4.5	62.8 ± 5.0				
Δ	−3.2 (−5.3, −1.1)**	0.9 (−1.3, 3.1)	−4.1 (−7.2, −1.0)	0.37	0.14	0.01

All baseline and 3–month values are unadjusted means ± SDs; all within-group changes are unadjusted mean (95% CI) and expressed as absolute change from baseline. Mean net differences were calculated by subtracting the within–group changes from baseline in the control group from the within–group change from baseline in the allo-HCT group after 3 months. *p < 0.05, **p < 0.01, ***p < 0.001 vs. baseline. BNP, brain natriuretic peptide; CI, cardiac index; cTn-I, troponin I; CMR, cardiac magnetic resonance imaging; GLS, global longitudinal strain; HR, heart rate; LVEF, left-ventricular ejection fraction; RVEF, right-ventricular ejection fraction; SVI, stroke volume index.

### Peak cardiac function and cardiac reserve

As shown in Table 4, allo-HCT was associated with a 13% reduction in CI_peak_ (*p* < 0.001) and a 9% reduction in SVI_peak_ (*p* = 0.003), but no change in HR_peak_. CI_peak_, SVI_peak_ and HR_peak_ remained unchanged in controls, resulting in a net group difference for the change at 3-months for CI_peak_ (interaction, *p* = 0.042), a trend toward a significant net group difference for SVI_peak_ (interaction, *p* = 0.058), but not HR_peak_ (interaction, *p* = 0.37). With respect to peak biventricular contractility, LVEF_peak_ and RVEF_peak_ remained unchanged in controls but decreased (absolute) 1.9% (*p* = 0.033) and 3.2% (*p* = 0.004), respectively, following allo-HCT, leading to a significant group-by-time interaction for RVEF_peak_ (*p* = 0.010), but not LVEF_peak_ (*p* = 0.14).

**Figure 2:**
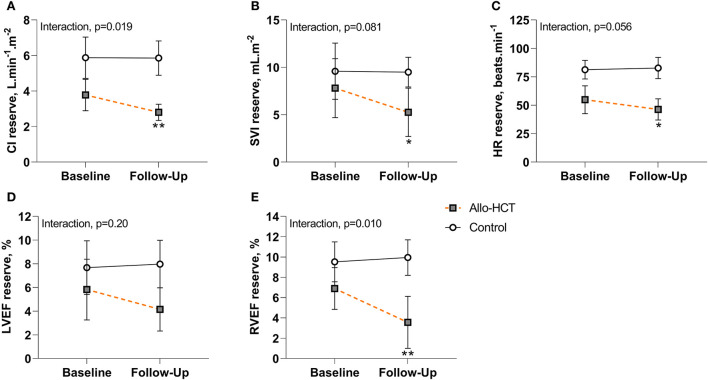
**(A–E)** Cardiac and contractile reserve at baseline and 3-month follow-up for Allo-HCT (*n* = 12) and Control (*n* = 11). After 3-months, cardiac and contractile reserve were maintained in controls, but allo-HCT experienced a blunted CI, SVI, HR, and RVEF reserve, resulting in a significant between-group difference for the net change from baseline for CI reserve and RVEF reserve and a trend toward a significant between-group difference for the net change from baseline for SVI reserve and HR reserve. *p < 0.05 and **p < 0.01 for within-group change in reserve. Data are unadjusted mean (95% CI). CI, cardiac index; HR, heart rate; LVEF, left-ventricular ejection fraction; RVEF, right-ventricular ejection fraction; SVI, stroke volume index.

Results pertaining to changes in cardiac and contractile reserve (ability to augment function above resting) are shown in Figures 2A–E. CI, SVI and HR reserve were unchanged in controls at 3-months, but were further blunted in allo-HCT recipients (*p* = 0.001, *p* = 0.010, *p* = 0.020, respectively), resulting in a net group difference for the change at 3-months for CI reserve (interaction, *p* = 0.019) and trends toward significant net group differences for SVI reserve and HR reserve (interaction, *p* = 0.081 and *p* = 0.056, respectively). A significant interaction was observed for RVEF reserve (*p* = 0.010), due to a blunted augmentation from rest to peak exercise in allo-HCT (*p* = 0.001) and no change in controls. There were no within- or between-group differences for LVEF reserve.

### Body composition and indices of vascular and hematological function

Weight declined 3.8 kg in allo-HCT recipients (*p* = 0.002), but this was not significantly different from the change in controls (−0.6 kg, *p* = 0.65; interaction, *p* = 0.082) (Table 5). Conversely, allo-HCT recipients experienced a significant net loss of 3.2 kg in LM relative to controls after 3-months (interaction, *p* = 0.001). No significant changes existed in either group for FM, %BF, hemoglobin, or BP. Three allo-HCT recipients and one control developed new-onset hypertension at 3-months, totaling a 35 and 8% prevalence, respectively (between-group difference, *p* = 0.19).

**Table 5:** Mean baseline values, within-group changes after 3–months and the net between-group differences for the change for body composition and indices of vascular and hematological function in Allo-HCT and Control groups.

**Measure**	**Allo-HCT**	**Control**	**Δ net difference**	**Group**,	**Time**,	**Group x Time**,
			**(HCT vs. Con)**	**p**	**p**	**p**
**Weight, kg**	
Baseline	80.7 ± 18.2	74.3 ± 14.4				
3–months	76.9 ± 18.1	73.6 ± 15.2				
Δ	−3.8 (−6.0, −1.5)**	−0.6 (−3.3, 2.1)	−3.2 (−6.7, 0.4)	0.48	0.016	0.082
**Body mass index, kg.m** ^**−2**^	
Baseline	27.4 ± 6.2	24.6 ± 3.7				
3–months	26.2 ± 6.3	24.4 ± 3.7				
Δ	−1.2 (−2.0, −0.5)**	−0.2 (−1.1, 0.7)	−1.0 (−2.2, 0.2)	0.26	0.014	0.090
**Total LM, kg**		
Baseline	51.7 ± 12.9	49.5 ± 10.1				
3–months	48.5 ± 11.9	49.5 ± 10.3				
Δ	−3.2 (−4.3, −2.0)***	0.0 (−1.4, 1.4)	−3.2 (−5.0, −1.3)	0.001	0.88	0.001
**Total FM, kg**		
Baseline	25.9 ± 10.3	21.8 ± 8.8				
3–months	25.3 ± 9.6	21.3 ± 8.1				
Δ	−0.6 (−2.7, 1.5)	−0.5 (−3.0, 2.0)	−0.1 (−3.4, 3.2)	0.25	0.48	0.95
**Body fat percentage, %**	
Baseline	31.8 ± 8.9	29.0 ± 8.8				
3–months	32.5 ± 8.5	28.6 ± 7.5				
Δ	0.7 (−1.4, 2.9)	−0.4 (−3.0, 2.2)	1.1 (−2.2, 4.5)	0.28	0.84	0.50
**SBP, mmHg**	
Baseline	129 ± 19	118 ± 15				
3–months	127 ± 20	118 ± 14				
Δ	−2 (−10, 6)	0 (−10, 10)	−2 (−14, 11)	0.071	0.78	0.80
**DBP, mmHg**	
Baseline	77 ± 15	70 ± 11				
3–months	77 ± 13	70 ± 11				
Δ	0 (−5, 5)	−1 (−7, 6)	1 (−7, 8)	0.11	0.90	0.90
**Hemoglobin, g.L** ^**−1**^	
Baseline	114.5 ± 20.0	138.9 ± 10.5				
3–months	107.2 ± 13.3	142.3 ± 9.7				
Δ	−7.3 (−16.5, 1.9)	3.4 (−8.6, 15.4)	−10.7 (−25.8, 4.4)	<0.001	0.61	0.16

All baseline and 3–month values are unadjusted means ± SDs; all within-group changes are unadjusted mean (95% CI) and expressed as absolute change from baseline. Mean net differences were calculated by subtracting the within–group changes from baseline in the control group from the within–group change from baseline in the allo–HCT group after 3 months. **p < 0.01, ***p < 0.001 vs. baseline. DBP, diastolic blood pressure; FM, fat mass; LM, lean body mass; SBP, systolic blood pressure.

## Discussion

To our knowledge, this is the first study to prospectively evaluate the short-term cardiovascular impact of allo-HCT among early transplant survivors, with comparison to an age-matched control group. Utilizing novel, state-of-the-art, non-invasive measures of cardiac function, this study demonstrated that, relative to matched controls, patients scheduled for allo-HCT presented with marked impairment in VO_2_peak and cardiac reserve. Importantly, we extend these findings and demonstrate that VO_2_peak and cardiac reserve deteriorate further in the 3-months following allo-HCT. Moreover, such impairments coincided with a reduction in a-vO_2_diff which makes an important contribution to the symptomatology of cardiovascular disorders such as heart failure (30). Collectively, these findings provide evidence of an accelerated cardiovascular aging phenotype that is present prior to transplant but is further exacerbated by the transplant and hospitalization process.

The inverse relationship between VO_2_peak and risk of cardiovascular morbidity, cardiovascular mortality, all-cause mortality, and cancer-specific mortality has been well established (25–27). In the present study, we observed a 26 and 24% reduction in absolute and bodyweight-indexed VO_2_peak in allo-HCT recipients over 3-months, which was ~9-fold greater than that observed in age-matched controls and approximates the degree of cardiovascular aging expected over 24 years of normal aging (38). While the 5.4 ml.kg^−1^.min^−1^ decline in VO_2_peak in allo-HCT recipients is profound, the true vulnerability of this population becomes especially evident when viewed in the context of the already diminished cardiovascular function prior to undergoing allo-HCT. Indeed, evidence from large prospective studies in ostensibly healthy non-cancer populations demonstrates that for each 1 MET (3.5 ml.kg^−1^.min^−1^) decrement in VO_2_peak, the risk of incident heart failure and all-cause mortality increases 16–21 and 25%, respectively (25, 39). In the present study, allo-HCT recipients achieved a VO_2_peak at follow-up that was 50% (16.4 ml.kg^−1^.min^−1^ or 4.7 METs) lower than controls, but of clinical relevance is that 53% of allo-HCT recipients were classified as being functionally disabled (VO_2_peak <18 ml.kg^−1^.min^−1^) at 3-months. This threshold has been associated with a reduced capacity to independently perform activities of daily living (40), and serves as a strong prognostic threshold, below which, the risk of incident heart failure and all-cause mortality are heightened 7-to-9-fold (26, 36). Notably, cross-sectional evaluation of VO_2_peak among long-term allo-HCT survivors (median time since allo-HCT, 9.8 years [range, 3−20]) indicates that these deleterious impairments in VO_2_peak do not fully recover over time, remaining substantially lower than predicted (22% below predicted) (7). Taken together, and consistent with the increased cardiovascular burden reported among long-term survivors (5–7, 9–12), these findings infer that allo-HCT recipients face a substantially greater risk of developing CVD relative to controls and provide novel insight into the potential trajectory of cardiovascular dysfunction in this cohort.

Dissecting the cardiac contribution to these reductions in VO_2_peak is critical given the potential role for pharmacological and non-pharmacological (i.e., lifestyle) interventions to attenuate cardiotoxicity. Using state-of-the-art exercise CMR, we provide the first evidence that treatment with allo-HCT significantly blunts cardiac reserve. Indeed, compared to pre-transplant, allo-HCT recipients experienced a blunted increase in SVI and HR from rest to peak exercise, resulting in a reduced augmentation in CI during exercise. These changes are indicative of myocardial injury/maladaptation—likely ascribed to both direct (i.e., cardiotoxic conditioning regimens) and indirect (i.e., physical inactivity, sedentary behavior) pathological perturbations (15, 16). The exact pathological mechanisms of allo-HCT induced cardiotoxicity are incompletely understood, but growing evidence suggests chemotherapy, radiotherapy, physical inactivity, and the allograft itself (by way of alloreactive donor T cell mediated immune and pro-inflammatory cytokine activation) can perturb the redox and inflammatory balance, which would theoretically lead to DNA damage, mitochondrial dysfunction, impaired sarcoplasmic reticulum calcium uptake activity, and extracellular matrix remodeling (i.e., fibrosis), and ultimately contractile dysfunction and cardiomyocyte apoptosis (41–44). The “mechanical unloading” associated with physical inactivity and bedrest may further compromise cardiac output *via* deconditioning of the cardiac muscle or *via* reductions in venous return and therefore stroke volume (45, 46). Moreover, it is important to highlight these declines in cardiac reserve induced by allo-HCT occurred on top of a reserve that was already diminished at baseline, such that following allo-HCT, CI_peak_, LVEF_peak_ and RVEF_peak_ were 26%, 7% (absolute), and 3% (absolute) lower than age-matched controls. Given that an inability to generate sufficient cardiac output during periods of high metabolic demand is an early hallmark of heart failure (24, 47), extrapolating these results over the years following allo-HCT—wherein normal age-related decline in cardiac function continues—may offer a possible explanation for the heightened prevalence of premature CVD and associated cardiovascular events and mortality in long-term survivors. Another intriguing finding from our study was that these allo-HCT induced reductions in cardiac reserve ensued whereas standard resting measures of cardiac function (LVEF, GLS, cardiac biomarkers) were unchanged. Indeed, echocardiographic parameters remained, on average, within normal ranges, and interpreted as an isolated assessment, would not flag an increased risk of CVD. These results are consistent with that observed among long-term allo-HCT survivors (normal LVEF despite impaired VO_2_peak at a median of 9.8 years after allo-HCT) (7) but are in contrast to Moriyama et al. (48) whom detected significant left-ventricular systolic dysfunction (characterized as a decrease in LVEF of ≥10% or LVEF ≤ 53%) in 17% of patients within 100 days after allo-HCT. These discrepant echocardiographic observations may be explained by differences in study design and potential selection bias. Indeed, we conducted a prospective echocardiographic assessment of all allo-SCT recipients whereas Moriyama et al. (48) conducted a retrospective review of allo-SCT recipients who underwent echocardiographic assessment at physician discretion (136/416 patients), biasing the likelihood of a cardiac finding. Nonetheless, the results of our study are consistent with the pattern of cardiac impairment seen among heart failure and anthracycline-treated cancer patients, wherein reductions in cardiac reserve often precede impairment in resting function (22–24, 47). Therefore, whilst not the primary aim of this study, our results also highlight the added utility of exercise cardiac reserve assessment in unmasking early treatment induced cardiac dysfunction in vulnerable populations.

Importantly, given non-cardiac factors also make important contributions to VO_2_peak (28) and the CVD phenotype (29–31), we explored whether the reduction in VO_2_peak observed among allo-HCT recipients could also reflect impairment in non-cardiac factors that determine peripheral muscle O_2_ delivery and utilization. In the present study, deficits in O_2_ carrying capacity as a result of anemia due to disease and prior therapies likely contributed to the baseline deficit in VO_2_peak among allo-HCT recipients (relative to controls) (49), but any further reductions in hemoglobin induced by allo-HCT had recovered at 3-months, and is therefore unlikely to explain the allo-HCT induced decline in VO_2_peak. We did, however, observe a significant reduction in peak a-vO_2_diff among allo-HCT recipients at 3-months. This is an important and novel finding as the impact of allo-HCT on skeletal muscle oxygenation has not been fully appreciated but may also contribute to the premature development of CVD and functional impairment in this population. Delineating the contribution of vascular and skeletal muscle factors to this decline in a-vO_2_diff will provide important insight into its clinical significance.

Premature vascular aging in allo-HCT patients may have been expected based on evidence from small cross-sectional and prospective studies which have evaluated vascular structure and function in the allo-HCT setting. Indeed, allo-HCT recipients have been shown to exhibit increased endothelial damage and dysfunction (evidenced by elevated circulating endothelial cells and soluble markers of endothelial damage, and lower endothelial dependent flow-mediated dilation) (50–53), central arterial stiffening (evidenced by increased aortic pulse wave velocity and reduced carotid distensibility, compliance and incremental elastic modulus) (54–56), and carotid intima-media thickening (54) compared to age-matched healthy controls or pre-transplant values. Consequently, hypertension is a common early (1-month incidence: 38–61%) (57, 58) and persistent complication of allo-HCT (odds ratio: 3.65 [95% CI, 1.82–7.32] at 8.6 years after allo-SCT) (59). It was therefore somewhat unexpected that the baseline prevalence and 3-month incidence of hypertension was similar between allo-HCT recipients and controls. This discrepancy could reflect the comparatively lower occurrence of grade II-IV GvHD and subsequent immunosuppressant exposure in our study (17, 18). Beyond this, it is important to note that subclinical vascular damage (i.e., endothelial dysfunction, arterial stiffness, intimal thickening) often precedes the development of hypertension and can remain “silent” for years before manifesting clinically, and therefore cannot be excluded as a possible mediator of allo-HCT-induced impairments in a-vO_2_diff and subsequently VO_2_peak. Moreover, there is emerging evidence that impairments in a-vO_2_diff, and subsequent exercise capacity following allo-HCT are explicable by concomitant skeletal muscle atrophy and mitochondrial dysfunction (60, 61). Indeed, as per-stated, generation of reactive oxygen species is a common effect of allo-HCT conditioning (41), allografting (43), and associated physical inactivity which can perturb homeostatic control of energy balance, upregulate muscle proteolytic and apoptotic signaling pathways, downregulate mitochondrial biogenesis and quality control pathways, and induce mitochondrial dysfunction (62). These deleterious processes may be further exacerbated by indirect treatment effects such as reductions in physical activity and dietary intake (63). Mitochondrial function was not directly assessed in the present study, but we did observe a significant reduction in LM which is a key determinant of VO_2_peak and risk factor for CVD (29). Taken together, with the cardiac insults, our results draw attention to the global nature of allo-HCT induced cardiovascular toxicity and highlights the need for cardiovascular preventive therapies capable of preserving and/or augmenting both central and peripheral determinants of VO_2_peak.

The strengths of this study include the prospective design, inclusion of a control group and the comprehensive cardiovascular evaluations employed which facilitated a more detailed characterization of the global cardiovascular consequences of allo-HCT than previously documented. A key limitation of the present study is the small cohort size which increases the possibility of type II error and precluded analyses of treatment-related and demographic modifiers of VO_2_peak and organ-specific function in our allo-SCT group. Such factors, particularly the impact of conditioning intensity (myeloablative vs. reduced intensity) which presumably impact the degree of cardiovascular damage incurred, warrant investigation in larger studies. Additionally, whilst we may speculate on the evolution of these changes in the years following allo-HCT, the short-term nature of this study limits our ability to explicitly discern the degree to which the observed changes depict persistent cardiovascular dysfunction that may culminate in overt CVD. Longitudinal assessment of these effects over subsequent years will be integral to understand their clinical trajectory and potential clinical significance. The selection of a cancer-free control group may be considered a limitation, however, the challenges associated with recruiting a suitable comparator should be acknowledged. Indeed, whilst ideal, it is implausible to compare to patients with similar hematological malignancies without allo-SCT due to the severity of the underlying illness and need for active treatment. From an alternate perspective, the inclusion of a cancer-free control group effectively highlights the pathological nature of the observed changes seen among allo-SCT recipients and provides important context of the true vulnerability of this high-risk patient group. Finally, we cannot exclude the possibility of subclinical allo-SCT induced vascular toxicity. A more detailed characterisation of effects of allo-HCT on subclinical vascular damage (e.g., endothelial dysfunction, arterial compliance) is required to provide a more complete understanding of the mechanisms underscoring the reduced VO_2_peak.

In summary, treatment with allo-HCT was associated with a marked reduction in VO_2_peak, reflecting a deterioration in both exercise cardiac reserve and a-vO_2_diff. Considering the inverse association between VO_2_peak and CVD risk, our results suggest that allo-HCT is a potent accelerator of cardiovascular aging, and provides valuable insight into the potential trajectory and pathogenesis of CVD in allo-HCT survivors. Combining these results with the existing cardiovascular dysfunction identified pre-allo-HCT, our study highlights the urgent need for preventive interventions—initiated early in, or even prior to, the allo-HCT process and capable of targeting the heart and periphery—to mitigate cardiovascular dysfunction in this high-risk patient group.

## Data availability statement

The reported data will be shared upon reasonable request to the corresponding author.

## Ethics statement

This study was reviewed and approved by the Alfred Hospital Ethics Committee. All experimental procedures conformed to the ethical standards set by the Helsinki Declaration. The patients/participants provided their written informed consent to participate in this study.

## Author contributions

EH, BK, DD, ALG, and SA contributed to the conceptualization and design of the study. HD, SF, YH-O, EH, and DK were responsible for participant recruitment. HD, SF, YH-O, and EH conducted data collection. HD, ALG, SF, and EH were responsible for image analyses (DXA, Echo, and CMR). HD and RD conducted the statistical analysis. HD drafted the original manuscript. All authors critically revised the manuscript and approved the final version.
