# Supplementary material for: Rapid cardiovascular aging following allogeneic hematopoietic cell transplantation for hematological malignancy
Source: Front Cardiovasc Med. 2022 Dec 15;9:926064. doi: 10.3389/fcvm.2022.926064 (PMC9797839; doi:10.3389/fcvm.2022.926064)
Supplement: Supplementary file 1 [file Table_1.docx]

**Supplementary Table 1**. Baseline and transplant characteristics of allo-HCT recipients who did and did not return for follow-up assessments.

|  | Returned  (n=17) | Did not return (n=9) | P |
| --- | --- | --- | --- |
| Sex, *% Male* | 70% | 56% | 0.53 |
| Age, *years* | 45 ± 18 | 49 ± 13 | 0.53 |
| Height, *m* | 1.72 ± 0.89 | 1.72 ± 0.10 | 0.97 |
| Weight, *kg* | 80.7 ± 18.0 | 89.7 ± 23.0 | 0.28 |
| Body mass index, *kg.m^-2^* | 27.5 ± 6.2 | 30.1 ± 5.6 | 0.29 |
| Hemoglobin, *g.L^-1^* | 114.5 ± 20.0 | 106.1 ± 18.9 | 0.33 |
| Cardiovascular Function |  |  |  |
| SBP, mmHg | 129 ± 19 | 127 ± 15 | 0.80 |
| DBP, mmHg | 77 ± 15 | 75 ± 11 | 0.67 |
| LVEF, % | 55 ± 5 | 58 ± 6 | 0.19 |
| GLS, % | -18 ± 2 | -19 ± 2 | 0.09 |
| CI Reserve, L.min^-1^.m^-2^ | 3.8 ± 1.4 | 3.2 ± 1.1 | 0.42 |
| VO_2_peak, ml.kg^-1^.min^-1^ | 22.9 ± 8.0 | 19.3 ± 7.0 | 0.32 |
| VO_2_peak, % predicted | 67 ± 12 | 71 ± 22 | 0.58 |
| Functional disability, n (%) | 5 (29%) | 6 (67%) | 0.10 |
| Cardiovascular Risk Factors, *n (%)* |  |  |  |
| Hypertension | 4 (24%) | 1 (11%) | 0.62 |
| Hyperlipidaemia | 2 (12%) | 0 (0%) | 0.53 |
| Diabetes | 1 (6%) | 0 (0%) | >0.99 |
| Body mass index ≥25kg.m^-2^ | 9 (53%) | 6 (67%) | 0.68 |
| Previous cardiovascular event | 2 (12%) | 0 (0%) | 0.53 |
| ≥1 cardiovascular risk factor | 10 (59%) | 6 (67%) | >0.99 |
| Cardiovascular Medications, *n (%)* |  |  |  |
| Statin/Cholesterol absorption inhibitor | 1 (6%) | 0 (0%) | >0.99 |
| Antihypertensives | 2 (12%) | 0 (0%) | 0.53 |
| Beta-blocker | 1 (6%) | 0 (0%) | >0.99 |
| Calcium channel blocker | 0 (0%) | 1 (11%) | 0.35 |
| Antidiabetic | 1 (6%) | 0 (0%) | >0.99 |
| NSAID | 3 (18%) | 0 (0%) | 0.53 |
| Diagnosis, *n (%)* |  |  |  |
| Acute lymphoblastic leukemia | 2 (12%) | 1 (11%) | >0.99 |
| Acute myeloid leukemia | 11 (65%) | 6 (67%) | >0.99 |
| Non-Hodgkin Lymphoma | 3 (18%) | 0 (0%) | 0.53 |
| Myelodysplasia | 1 (6%) | 1 (11%) | >0.99 |
| Severe aplastic anaemia | 0 (0%) | 1 (11%) | 0.35 |
| Prior Cancer Treatment, *n (%)* |  |  |  |
| No Prior Treatment | 1 (6%) | 1 (11%) | >0.99 |
| Radiation | 2 (12%) | 0 (0%) | 0.53 |
| Targeted Therapy | 5 (29%) | 3 (33%) | >0.99 |
| Immunotherapy | 3 (18%) | 0 (0%) | 0.53 |
| Autologous stem cell transplant | 1 (6%) | 0 (0%) | >0.99 |
| Chemotherapy | 16 (94%) | 8 (89%) | >0.99 |
| Anthracyclines | 14 (82%) | 7 (78%) | >0.99 |
| *Cumulative anthracycline dose, mg.m^-2^* | 180 (100-270) | 270 (90-315) | 0.85**^α^** |
| Anti-Metabolites | 14 (82%) | 7 (78%) | >0.99 |
| Alkylating Agents | 5 (29%) | 1 (11%) | 0.38 |
| Plant Alkaloids | 10 (59%) | 4 (44%) | 0.68 |
| Miscellaneous Antineoplastics | 1 (6%) | 1 (11%) | >0.99 |
| Donor Type, *n (%)* |  |  |  |
| Related | 8 (47%) | 3 (33%) | 0.68 |
| Unrelated | 9 (53%) | 6 (67%) | 0.68 |
| Graft Source, *n (%)* |  |  |  |
| Bone Marrow | 3 (18%) | 1 (11%) | >0.99 |
| Peripheral Blood Stem Cell | 14 (82%) | 7 (78%) | >0.99 |
| Cord Blood | 0 (0%) | 1 (11%) | 0.35 |
| Conditioning Intensity, *n (%)* |  |  |  |
| Myeloablative | 7 (41%) | 8 (89%) | 0.036 |
| Reduced Intensity | 10 (59%) | 1 (11%) | 0.036 |
| Conditioning Regimen, *n (%)* |  |  |  |
| Cy | 0 (0%) | 1 (11%) | 0.35 |
| Cy/TBI | 6 (35%) | 5 (56%) | 0.42 |
| Cy/Flu/TBI/TT | 0 (0%) | 1 (11%) | 0.35 |
| Flu/Mel | 6 (35%) | 2 (22%) | 0.67 |
| Flu/Mel/Campath | 3 (18%) | 0 (0%) | 0.53 |
| ETP/TBI | 1 (6%) | 0 (0%) | >0.99 |
| LACE | 1 (6%) | 0 (0%) | >0.99 |
| GvHD Prophylaxis, *n (%)* |  |  |  |
| MTX/Ciclosporin ± ATG | 10 (59%) | 6 (67%) | >0.99 |
| PTCy/Ciclosporin | 4 (24%) | 1 (11%) | 0.63 |
| Ciclosporin ± ATG | 2 (12%) | 1 (11%) | >0.99 |
| Ciclosporin/Myco | 0 (0%) | 1 (11%) | 0.35 |
| TAC | 1 (6%) | 0 (0%) | >0.99 |
| Acute GvHD Grade, *n (%)* |  |  |  |
| No GvHD | 10 (59%) | 6 (67%) | >0.99 |
| Grade I | 6 (35%) | 0 (0%) | 0.06 |
| Grade II | 1 (6%) | 0 (0%) | >0.99 |
| Grade IV | 0 (0%) | 2 (22%) | 0.11 |
| Hospital Length of Stay, days | 31 (27-35) | 36 (26-53) | 0.43**^α^** |
| Data are mean ± SD, median (IQR) or n (%). ^α^ assessed by Mann-Whitey *U* test. Abbreviations: CI, cardiac reserve; Cy, Cyclophosphamide; DBP, diastolic blood pressure; ETP, Etoposide; Flu, Fludarabine; GLS, global longitudinal strain; GvHD, Graft versus host disease; LACE, Lomustine, Cytarabine, Cyclophosphamide, Etoposide; LVEF, left-ventricular ejection fraction; Mel, Melphalan; MTX, Methotrexate; Myco, Mycophenolate; PTCy, Post-Transplant Cyclophosphamide; TAC, Tacrolimus; SBP, systolic blood pressure; TBI, Total body irradiation; TT, Thiotepa; VO_2_peak, peak oxygen uptake. | | | |
